# Editorial: Controversies in the Local Management of Lung Cancer

**DOI:** 10.3389/fonc.2018.00233

**Published:** 2018-06-18

**Authors:** John M. Varlotto, Giulia Veronesi

**Affiliations:** ^1^Department of Radiation Oncology, University of Massachusetts Medical Center, Worcester, MA, United States; ^2^Division of Thoracic Surgery, Humanitas Clinical and Research Center, Rozzano, Milan, Italy

**Keywords:** lung cancer, multi-modality treatment, thoracic surgery, radiation therapy, lung cancer treatment

**Editorial on the Research Topic**

**Controversies in the Local Management of Lung Cancer**

At the time we agreed to edit this special edition of Frontiers in early 2017, the immunotherapy revolution was already established in the second-line treatment of metastatic non-small cell lung cancer (NSCLC) (1–4) and even to a select group of patients (those whose tumor cells expressed PD-L1 of ≥50%) (5) in the first-line treatment of metastatic disease. We felt that the summarization and understanding of the strengths and benefits of our local modalities were needed prior to the possible integration of immunotherapy into the treatment paradigms of Stages I–III NSCLC and small cell lung cancer (SCLC).

Three of this issue’s 10 article deal with the controversial topic of neo-adjuvant treatment prior to surgery in the management of Stage III lung cancer from the prospective of a multi-disciplinary team (Lewis et al.), a Radiation Oncologist (Sher), and a Thoracic Surgical group (Van et al.). Although no definitive management strategy was recommended as “the way” to manage Stage III NSCLC, each article does a great job of reviewing the current literature, while offering its own unique perspective. The article by Van et al. additionally discusses the rarely investigated topic of salvage surgery.

Four articles dealt with unconventional and/or innovative uses of radiation. Ohri provides a provocative article concerning dose escalation as well as dose de-escalation. Although the idea of dose de-escalation is unpalatable to most Radiation Oncologists, this idea may be considered to be prescient because of the now described synergism between concomitant cytotoxic therapy and immunotherapy in patients with metastatic non-squamous cell NSCLC (6). Kumar et al. offer a pioneering approach to the treatment of Stage III NSCLC which they have labeled quadmodality therapy (concurrent chemo/radiation followed by a stereotactic boost to residual sites of disease, followed by immunotherapy) for the more aggressive treatment of locally advanced lung cancer (Kumar et al.). Both studies, offer different approaches to the hopefully successful integration of immunotherapy with standard chemo/radiation in the near future. Bergsma et al. discuss the rationale and increasingly strong evidence for administering stereotactic body radiation therapy (SBRT) in patients with oligometastatic NSCLC (Bergsma et al.). The work of these investigators and others have led to the NRG LU002 trial that will be investigating the role of SBRT for patients with three or fewer sites of remaining extracranial disease after chemotherapy. Chun et al. have provided a nice review article on heavy ion therapy in the management of NSCLC. Although the recently published prospective randomized trial of passive scattering proton radiation vs intensity-modulated radiation did not reveal any benefit from proton therapy in terms of local failure, radiation pneumonitis, and lung dosimetry, protons resulted in lower heart doses despite larger having larger gross tumor volumes in that trial arm (7). Because heart dose was a strong determinant of overall survival in RTOG 0167’s failure to improve outcomes by radiation dose escalation (8), it is hoped that the Bragg–Peak associated with heavy ion therapy will be proven to offer improved local control while limiting normal tissue damage.

The articles by Bloom et al. and Jeremic et al. remind the readers of Frontiers how important local management is to the outcomes of SCLC. Bloom et al. offer a comprehensive review of the literature concerning prophylactic cranial irradiation (PCI) in the management of surgically resectable SCLC. Because the incidence of early-stage SCLC is increasing (9), the review of this topic is very important. Although the authors could not reach a definitive conclusion regarding PCI, it is hoped that NRG CC003 demonstrates that hippocampal sparing allows for effective PCI while limiting harmful neurocognitive sequelae and makes the decision to administer PCI in surgically resectable SCLC an easier one. Jeremic et al. comprehensively review consolidative thoracic radiation in the management of extensive stage SCLC from their pioneering work published in 1999 (10) to the more recent work of Slotman et al. (11). They also discuss the potential merits of different radiation fractionation regimens and the use of sequential vs concomitant chemo/radiation regimens. Furthermore, the authors provide a timely update of the conflicting data (12, 13) in regards to the use of PCI for the treatment of patients with extensive stage SCLC.

Although there are no articles in this edition concerning the benefits and risk of SBRT in comparison to surgical resection for patients with Stage I NSCLC, and past retrospective studies (14, 15) and one small publication of prospective data (16) have suggested equivalency, it is hoped that future studies may shed light on proper patient selection for either of these very effective techniques. Until then, the article by Varlotto et al. sheds needed light on how psychosocial aspects can adversely affect the outcomes of surgically resected NSCLC. These authors demonstrated that not being married and having insurance resulted in lower overall survival/lung cancer-specific survival, increased 90-day mortality and a higher incidence of positive nodes upon resection.

During the last decade surgical treatment of lung cancer changed deeply thanks to the large diffusion of minimally invasive approaches that become the standard approach for early-stage lung cancer (17). Thanks to the introduction of robotic technology, more recently, even selected patients with more advanced disease, even after induction treatment for N2 involvement, have been approached with minimally invasive robotic surgery to reduce surgical trauma, a very important goal in patients with already increased fragility due to systemic treatment (18). Recent publications showed that robotic approach was safe and effective in this subgroup of patients, paving the way to potential changes in the indications of surgical resection in stage III patients, and a rethinking of the better timing for systemic and local treatment (19) with potential advantages to propose minimally invasive surgery upfront for N2 single station disease.

Since our initial agreement to edit this special edition, immunotherapy has continued to radically change the lung cancer landscape. A press release from Merck on April 9, 2018 concerning Keynote-042 demonstrated a superior OS for pembrolizumab (a PD-1 inhibitor) as compared to platinum-based chemotherapy in the first-line setting. The benefit was shown in a population of patients with PD-L1 of ≥1% which means that the majority of patients presenting with metastatic NSCLC can benefit from immunotherapy alone as their initial treatment. Furthermore, Keynote 189 (Van et al.) noted an improved overall survival and progression-free survival using combined immunotherapy (pembolizumab) and chemotherapy as compared to chemotherapy alone. Additionally, consolidative durvalumab (a PD-L1 inhibitor) has shown an impressive progression-free survival benefit in patients with stage III lung cancer after the completion of concurrent chemo/radiation (20). We feel that the articles in this special edition can help the readers of Frontiers better understand the strength and limitations of our existing local therapies so that we can better understand how to incorporate immunotherapy in the management of Stage I–III NSCLC and SCLC.

We are very grateful to the tremendous efforts of the fellow authors, the reviewers, and staff at Frontiers for making this special edition possible.

## Author Contributions

GV and JV both wrote this article.

## Conflict of Interest Statement

The authors declare that the research was conducted in the absence of any commercial or financial relationships that could be construed as a potential conflict of interest.
